# Chagas disease (American trypanosomiasis) in the context of the 2030 agenda. Insights into global warming and vector control

**DOI:** 10.1590/0074-02760210479chgsb

**Published:** 2022-05-27

**Authors:** José Jurberg

**Affiliations:** 1 Instituto Oswaldo Cruz-Fiocruz

Although it has been 112 years since the important discovery that a blood-sucking insect could transmit a parasite to humans and cause a disease, in the 21st century we are still facing the same problems raised at that time. According to Souza and her fellow researchers,^1^ despite the successful implementation of some control programs in many countries, which led to a substantial decrease in the transmission rate of *Trypanosoma cruzi*, the still existing precarious housing conditions, the absence of proper sanitation, health programs with limited coverage, and food insecurity contribute to results that are still unsatisfactory in certain nations. Faced with climate change, social inequalities, and migration flows, among other challenges, these countries also have to deal with exponential population growth without the support of an adequate policy related to economic, social and environmental aspects. This hinders the design, development and integration of efforts aimed at vector control and transmission of Chagas disease as discussed in the 2030 Agenda for Sustainable Development.^1^

As the authors state in their article, “the world is opening up to the renewal of surveillance practices, but the lessons learned in the past should be the basis of solutions for the future.” Thus, I bring insights from the past to think about the present and the future control of Chagas disease in Brazil and perhaps in other nations.

Carlos Chagas’s first demonstration of what was thought to be a new disease showed a mud hut and people lacking minimum sanitation conditions, water supply, and sewage systems. In a nutshell, a depiction of poverty and hunger. Unfortunately, the same scenario persists and worsens with the oral transmission that accounts for most of the records of acute infections in Brazil.^2^ One does not have to go to the outskirts of the country to find, still today, thousands of houses/huts with the same characteristics of that time and a completely unassisted population.

In 1909, given the difficulty in identifying that bug, scientist Arthur Neiva was appointed to the task that seemed extremely challenging. This marked the onset of the implementation of what would become the School of Triatomines at the Oswaldo Cruz Institute. The bug was initially classified as *Conorhinus megistus* Burm, then renamed *Panstrongylus megistus* Burmeister. Interest in the discovery caught the attention of Cesar Pinto, another Oswaldo Cruz Institute researcher who also began to devote himself to the study of triatomines. Neiva and Pinto were the first generation of researchers dedicated to the subject. The second generation included Herman Lent, in 1935, who was also dedicated to studying the insect vectors of Chagas disease until 1970, when he was compulsorily retired by the Military Dictatorship in 1964, as he described in his memoirs, “O massacre de Manguinhos” (The Manguinhos Slaughter).

It was Lent who created and kept the insectary, which is currently considered one of the largest in the world, with more than 45 species in 150 crystallisers. Since then, the insectary has supplied identified insects - dead or alive - at the request of interested health professionals. In addition, Lent broke ground by implementing a collection of 24,000 specimens, most of which have been identified and registered, and are currently being digitised. This collection consists of material collected by all those interested in Triatomines. There were some 9,000 specimens, which later received an additional 15,000 specimens from the collection of Argentine researcher Carcavallo.^3^

As discussed by Souza et al.,^1^ the control results have been variable to date. Taxonomy has significantly developed there after I joined the laboratory in 1960, since, together with Herman Lent, we introduced a new approach to identifying Triatomines: the comparative study focused on phallic structures.^4^ ^,^ ^5^ ^,^ ^6^ ^,^ ^7^ In addition, many other researchers have brought new approaches to the subfamily Triatominae to date, resulting in 18 genera and more than 150 species addressed in hundreds - if not thousands - of articles published since 1909.

The new analytical tool targeted at the phallic organ^4^ ^,^ ^5^ ^,^ ^6^ ^,^ ^7^ showed that there are 20 structures in different shapes for each species and the presence or absence of the structure designates their genera.

Dedicated to the classification of triatomines over the course of six decades, I have come across the guidelines of the 2030 Agenda mentioned by Souza et al.,^1^ which made me think that, unfortunately, we are still far from protecting the people and the planet, and from ensuring peace and prosperity through partnerships. In our scientific microenvironment, we seek to develop a new approach by using education to control these bugs and prevent Chagas disease. In this context, we have developed a number of tools to accomplish this purpose, and we have enabled, as I see it, easier conditions to control the triatomine fauna of the various Brazilian regions and also of Spanish-speaking countries. Through the dissemination and popularisation of knowledge, we have put out a collection of five mini-notepads with colored images of triatomines found in each Brazilian region, i.e., North, Northeast, South, Southeast and Midwest.^8^ In addition, we have designed and distributed an illustrated booklet with the natural history of triatomines; an Iconographic Atlas^3^ ^,^ ^9^ and several book chapters on triatomines. All this content is freely offered to interested parties, including researchers, public and private secondary and elementary school teachers,^10^ health agents, librarians and students.

The use of this teaching material has brought about a significant change to vector control, as it explains the role of the bugs in the spread of the disease, as noted by health agents. This has led to a decrease in the number of requests to identify insect vectors of Chagas disease, turning, in my opinion, small initiatives that involve education and access to science into what Souza et al.^1^ mention as innovation with communities and cooperation aimed at long-term results, with support from local leaders.

Much has been - and continues to be - done for vector control. In their article, Souza et al.^1^ set a timeline on the effective use of insecticides to eliminate triatomines and the effective success and failure of initiatives to control Chagas disease in Brazil. Looking at the past and looking forward in terms of disease control, both in Brazil and other countries, I still consider this a huge challenge that will only be overcome when we actually have less inequality and more investments in economic, social and environmental areas, as also pointed out by Souza et al.,^1^ by bringing together a comprehensive approach that includes several aspects of social improvement, such as access to suitable housing with the sustainable management of water, sanitation and energy; ensured food security; equitable and quality education; healthy life; inclusive economic growth with suitable employment, work and income, among others. In short: an actual people’s protection policy, as mentioned in the Agenda 2030 action plan.

Otherwise, 14 out of the 17 goals within the sustainable development goals (SDGs) listed by Souza et al.^1^ will be ineffective, and we could describe a scenario for 2030 that may reach, rather than the 70 million people at risk in the world, as current estimates indicate, 140 million or more individuals affected by the disease, with the poorest and unassisted being the most vulnerable ones.

Comments on the article: Souza RCM, Gorla DE, Chame M, Jaramillo N, Monroy C, Diotaiuti L. Chagas disease in the context of the 2030 agenda: global warming and vectors. Mem Inst Oswaldo Cruz. 2022; 117: e200479.
